# Evolution and Biogeography of the Slipper Orchids: Eocene Vicariance of the Conduplicate Genera in the Old and New World Tropics

**DOI:** 10.1371/journal.pone.0038788

**Published:** 2012-06-07

**Authors:** Yan-Yan Guo, Yi-Bo Luo, Zhong-Jian Liu, Xiao-Quan Wang

**Affiliations:** 1 State Key Laboratory of Systematic and Evolutionary Botany, Institute of Botany, the Chinese Academy of Sciences, Beijing, China; 2 Graduate University of the Chinese Academy of Sciences, Beijing, China; 3 The Orchid Conservation and Research Center of Shenzhen, Shenzhen, China; CNR, Italy

## Abstract

Intercontinental disjunctions between tropical regions, which harbor two-thirds of the flowering plants, have drawn great interest from biologists and biogeographers. Most previous studies on these distribution patterns focused on woody plants, and paid little attention to herbs. The Orchidaceae is one of the largest families of angiosperms, with a herbaceous habit and a high species diversity in the Tropics. Here we investigate the evolutionary and biogeographical history of the slipper orchids, which represents a monophyletic subfamily (Cypripedioideae) of the orchid family and comprises five genera that are disjunctly distributed in tropical to temperate regions. A relatively well-resolved and highly supported phylogeny of slipper orchids was reconstructed based on sequence analyses of six maternally inherited chloroplast and two low-copy nuclear genes (*LFY* and *ACO*). We found that the genus *Cypripedium* with a wide distribution in the northern temperate and subtropical zones diverged first, followed by *Selenipedium* endemic to South America, and finally conduplicate-leaved genera in the Tropics. *Mexipedium* and *Phragmipedium* from the neotropics are most closely related, and form a clade sister to *Paphiopedilum* from tropical Asia. According to molecular clock estimates, the genus *Selenipedium* originated in Palaeocene, while the most recent common ancestor of conduplicate-leaved slipper orchids could be dated back to the Eocene. Ancestral area reconstruction indicates that vicariance is responsible for the disjunct distribution of conduplicate slipper orchids in palaeotropical and neotropical regions. Our study sheds some light on mechanisms underlying generic and species diversification in the orchid family and tropical disjunctions of herbaceous plant groups. In addition, we suggest that the biogeographical study should sample both regional endemics and their widespread relatives.

## Introduction

Tropical regions harbor almost two-thirds of the flowering plants [1], [2], where intercontinental disjunctions occur commonly within and among plant genera due to Gondwana breakup, immigration from the Laurasian tropics and transoceanic dispersal [3], [4]. Compared with the Southern Hemisphere biogeography, whether vicariance or long distance dispersal has played a more important role during and after the fragmentation of Gondwana (160-30 Mya) [5], [6], biogeography of the Northern Hemisphere is more complex because of not only the impact of climatic and geological changes [7]–[9], but also the frequent migration by the North Atlantic land bridge and the Bering land bridge in the Tertiary [10]–[14]. A series of studies have suggested the boreotropical region as a corridor for the migration of thermophilic groups, such as Magnoliaceae [15], [16], Alangiaceae [17], Burmanniaceae [18], Altingiaceae [19], and Malpighiaceae [20]. However, most of them focused on woody plants, and paid little attention to herbs, which have shorter life histories, higher rates of molecular evolution [21], and much fewer fossils due to differential leaf and pollen production [22]. It would be of great interest to investigate the biogeographical history of herbaceous plant groups showing tropical disjunct distributions.

On the other hand, owing to the occurrence of a series of climatic oscillations and geographic events in the past 65 Mya [12], [13], [23]–[25], plants not only experienced expansion and contraction of their ranges [26]–[30], but also diversified to adapt to new niches [31]–[35]. It may explain why Wing [36] detected a mixture of tropical and temperate elements in the Eocene floras of the Rocky Mountains. Lavin & Luckow [37] and Wen [38] proposed that the study of disjunctions in temperate groups should include their subtropical and tropical relatives, and vice versa.

Orchidaceae is one of the largest families of flowering plants, accounting for approximately 10% of seed plants [39]. All orchids are herbaceous, of which about 73% are epiphytic or lithophytic [39]. According to fossil records, a fossil orchid with its pollinator in particular, the common ancestor of modern orchid lineages could be dated back to the late Cretaceous [40]–[42], although the radiation of most clades of the Orchidaceae occurred in the Tertiary. The subfamily Cypripedioideae (slipper orchids) is one of the monophyletic groups of Orchidaceae [43]–[48], including all the species with a pouchlike lip, two fertile stamens, a shield-like staminode and a synsepal composed of the fused lateral sepals [49]. There are almost 200 species of slipper orchids (http://apps.kew.org/wcsp/), belonging to five accepted genera, i.e., *Cypripedium*, *Mexipedium*, *Paphiopedilum*, *Phragmipedium* and *Selenipedium* [50]. The attractive flowers of slipper orchids make them have high ornamental and commercial values, and hold a special place in the hearts of botanists and hobbyists [51]. Also, this group is the most studied among all orchids due to its distinctive features [52]–[59]. Dressler [60] even considered that this group could have an unusual way of specialization given its unique flower morphology.

Pfitzer [61] and Atwood [62] investigated the relationships of slipper orchids based on morphological data, then Albert [63] based on both morphology and the chloroplast *rbc*L gene, and Cox et al. [64] using nuclear ribosomal DNA internal transcribed spacers (nrDNA ITS). Besides, several phylogenetic studies of Orchidaceae sampled slipper orchids [43], [46]–[48], [65]. All the previous studies strongly support the monophyly of slipper orchids, but have not reached a consensus about the intergeneric relationships, and in particular the published chloroplast DNA (cpDNA) phylogenies have low resolution or incomplete sampling in this orchid clade [43], [46], [47], [58].

**Figure 1:**
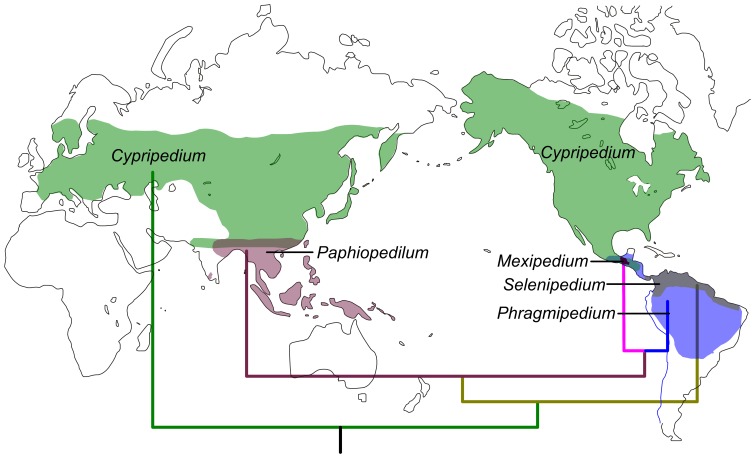
The distribution of slipper orchids modified from Pridgeon et al. [**165**]. Shaded areas show the current species distribution, with different colors to represent the five genera. The tree topology indicates the phylogenetic relationships of slipper orchids reconstructed in this study.

The slipper orchids are widely distributed in temperate to tropical regions of Eurasia and America. The genus *Cypripedium* occurs in temperate and subtropical areas of the North Hemisphere, with some species extending to tropical North America. The two conduplicate-leaved genera *Mexipedium* and *Phragmipedium* and the plicate-leaved genus *Selenipedium* are restricted to the neotropics, whereas *Paphiopedilum* is confined to the palaeotropics (Fig. 1). Atwood [62] and Albert [63] supported the boreotropical hypothesis [66], and considered that fragmentation of continents and the following climatic cooling in the Ice Ages caused the present disjunct distribution of slipper orchids. While the ITS analysis supports southern North America/Mesoamerica as the origin center of slipper orchids [64], the sister relationship between *Mexipedium* and *Paphiopedilum* revealed in the low copy nuclear *Xdh* gene phylogeny [48], although with weak support and based on a limited sampling, seems to suggest a long distance dispersal from palaeotropical to neotropical regions. Therefore, the biogeographical history of slipper orchids is far from being resolved.

It has been widely recognized that the use of multiple genes is helpful for the accuracy of phylogenetic and biogeographical reconstruction (e.g. [67], [68]). In addition to the widely used cpDNA markers such as *rbc*L, *mat*K, *ndh*F and *ycf*2 [69]–[72], more and more studies indicate that *ycf*1, one of the two longest coding genes of cpDNA, has great potential in plant phylogenetic reconstruction [73]–[75]. Meanwhile, single or low copy nuclear genes are increasingly used in plant phylogenetic studies due to their rapid evolutionary rates and biparental inheritance [76]–[80]. For instance, *LFY*, which is involved in regulating flower meristem identity and flowering time [81]–[84], has been successfully used as a single copy gene to investigate intra- and inter-generic relationships [68], [85]–[88], and allopolyploid speciation [89]. Also, the *ACO* gene, which encodes the *ACC* oxidase enzyme to catalyze the last step of ethylene biosynthesis in plants [90], is important for flower development, fruit ripening, and responses to biotic and abiotic stresses [91]. This gene may also exist as a single locus in slipper orchids according to the result of 3′-RACE.

In the present study, we aim to reconstruct the phylogeny of slipper orchids with multiple coding chloroplast and low copy nuclear genes. In addition, we intend to estimate divergence times of the five genera of slipper orchids, and to explore their biogeographical history, particularly the disjunction between neotropical and palaeotropical regions. This study may also shed some light on the mechanisms underlying the diversification of Orchidaceae.

## Materials and Methods

### Ethics statement

No specific permits were required for the described field studies.

### Plant sampling

We sampled 31 species, which represent all five genera of the subfamily Cypripedioideae and cover seven sections of *Paphiopedilum* and four sections of *Phragmipedium*. In the genus *Cypripedium*, 16 species of nine sections were collected from eastern Asia and North America. Owing to the rarity and difficulty in collection, this study only sampled one individual of *Selenipedium*, a genus with five accepted species that are morphologically similar and endemic to the tropical regions of Central and South America [92]. In addition, four species representing three genera of the two subfamilies Apostasioideae and Vanilloideae were chosen as outgroups, since previous studies showed that Apostasioideae and Vanilloideae are sister to slipper orchids plus the other monandrous orchids [44], [48]. The origins of the materials are shown in Supplementary Table S1.

### DNA extraction, PCR amplification, cloning and sequencing

Total DNA was extracted from silica gel-dried leaves using a modified cetyltrimethylammonium bromide (CTAB) protocol [93] or Plant Genomic DNA Kit (Tiangen Biotech Co.). We screened eight chloroplast coding genes (*acc*D, *rbc*L, *mat*K, *rpo*C1, *rpo*C2, ycf1, *ycf*2, and *ndh*F) and two low-copy nuclear genes (*LFY* and *ACO*). The *LFY* gene was amplified with the forward primer *LFY*E1jF (5′-TGAGGGAGGAGGAGGTSGACGAYATGAT-3′) located at the first exon and the reverse primer *LFY*E3kR (5′-AGATBGAGAGGCGSGGATGSGCGTT GAA-3′) at the third exon, and the *ACO* gene with *ACO*E1aF (5′-GCNTGYGAGAA CTGGGGHTTCTTYGAG-3′) and *ACO*E2aR (5′-ATGGTCTTCATGGCCTCAA ACCT-3′). All the four primers were designed based on the sequences available in the public databases. However, the two *LFY* primers did not work in *Phragmipedium besseae*, *Paphiopedilum delenatii*, *P. vietnamense*, and some species of *Cypripedium*, and thus another reverse primer *LFY*E2S5R at the second exon was further designed. The details of other primers are shown in Supplementary Table S2. Although the *ndh*F gene is conservatively located at the small single copy (SSC) region of the published chloroplast genomes, e.g. *Oryza sativa* [94], *Amborella trichopoda* [95], *Nymphaea alba* [96] and *Acorus calamus* [97], it was reported to have been lost in the sequenced chloroplast genomes of the four orchids *Phalaenopsis aphrodite* [98], *Oncidium* Gower Ramsey [99], *Rhizanthella gardneri* [100] and *Neottia nidus-avis* [101]. Therefore, we tried to amplify the *ndh*F gene with primers *trn*N_guu_ and *trn*L_uag_ that are located in its two flanking regions, and *ndh*FcF and *ndh*FaR in its coding regions, respectively.

Amplification reactions were conducted in a Tgradient Thermocycler (Biometra) or a Mastercycler (Eppendorf, Hamburg, Germany) in a volume of 25 µL containing 10–50 ng DNA template, 200 µmol/L of each dNTP, 6.25 pmol of each primer pair, and 0.75 U of Taq DNA polymerase (TakaRa Biotech Co., Dalian, China). PCR cycles are as follows: for the chloroplast genes, 4 min at 70°C, 4 cycles of 2 min at 94°C, 30 s at 51°C, and 1–3 min at 72°C, followed by 36 cycles of 30 s at 94°C, 30 s at 53°C, and 1–3 min at 72°C, with a final elongation for 10 min at 72°C; for the nuclear genes, 4 min at 70°C, 4 cycles of 2 min at 94°C, 30 s at 57°C, and 5 min at 68°C, followed by 36 cycles of 30 s at 94°C, 30 s at 60°C, and 5 min at 68°C, with a final extension for 15 min at 68°C. PCR products were separated by 1.5% agarose gel electrophoresis and purified with a Gel Band Purification Kit (TIANgel Midi Purification Kit). The purified PCR products of the chloroplast genes were directly sequenced with the PCR primers and the internal primers designed in this study (Supplementary Table S2). For the nuclear genes, the purified PCR products were cloned with pGEM-T® Easy Vector System II (Promega). Twelve clones were picked for each sample, and 4–6 of them with correct insertion (determined by digestion with *EcoR* I) were sequenced with primers T7 and SP6 and the internal primers designed in this study (Supplementary Table S2). After precipitation with 95% EtOH, 3 M NaAc and 125 mM EDTA, the sequencing products were separated on an ABI PRISM 3730XL DNA analyzer (Applied Biosystems). The sequences reported in this study are deposited in GenBank under accession numbers JN181400–JN181549 and JQ182152–JQ182298 (Supplementary Table S1).

### Data analysis

The ContigExpress program of the Vector NTI Suite 6.0 (Informax Inc.) was used to assemble sequences from different primers. Sequence alignments were made with BioEdit 7.0 [102] and refined manually. Nucleotide diversity (Pi) was estimated using DnaSP version 5.0 [103]. Indels were coded using GapCoder [104], with a ‘1’ for present, ‘0’ for missing, and ‘-’ for inapplicable. The unalignable regions of the *rpo*C1 intron were excluded from our analyses. The incongruence length difference (ILD) test [105] was used to assess the congruence between different datasets. Phylogenetic analyses based on maximum parsimony (MP), maximum likelihood (ML) and Bayesian inference (BI) were performed with PAUP version 4.0b10 [106], PhyML 2.4.4 [107] and MrBayes 3.1.2 [108], respectively. In the MP and ML analyses, the missing data were coded by “?”, while it was excluded from the BI analysis. The MP analysis used a heuristic search with 1000 random addition sequence replicates, tree-bisection-reconnection (TBR) and MULTREES on, and branch support was evaluated by bootstrap analysis [109] of 1000 replicates using the same heuristic search settings. The evolutionary models for the ML and BI analyses were determined by Modeltest 3.07 [110] and MrModeltest v2.2 [111], respectively (Table 1). The ML analysis used the GTR model and a BIONJ tree as a starting point, and branch support was estimated by bootstrap analysis [109] of 1000 replicates. For the Bayesian inference, one cold and three incrementally heated Markov chain Monte Carlo (MCMC) chains were run for 1,000,000 cycles and repeated twice to avoid spurious results. One tree per 100 generations was saved. The first 300 samples for each run were discarded as burn-in to ensure that the chains had become stationary. Phylogenetic inferences were made based on the trees sampled after generation 30,000.

**Table 1:** Results of Model test and MrModel test.

	Model test		MrModel test	
	AIC	hLRTs	AIC	hLRTs
**combined cpDNA**	TVM+I+G	TVM+I+G	GTR+I+G	GTR+I+G
***ACO***	K81uf+G	K80+G	GTR+G	SYM+G
***LFY***	GTR+G	TrN+G	GTR+G	GTR+G
**combined nuclear DNA**	GTR+G	GTR+G	GTR+G	GTR+G
**cpDNA+nuclear DNA**	TVM+I+G	TVM+I+G	GTR+I+G	GTR+I+G

Molecular dating is very helpful to interpret plant distribution patterns [112], [113]. The likelihood ratio test (LRT) was used to test the rate constancy among lineages [114]. Log likelihood ratios of the chosen model with and without an enforced molecular clock were compared. The degree of freedom is equivalent to the number of terminal taxa minus two [115]. Significance was assessed by comparing two times the log likelihood difference to a chi-square distribution. Due to the lack of fossil evidence for the subfamily Cypripedioideae, we first performed a family-level analysis to get a more reliable estimate of the divergence times in Orchidaceae by integrating all the three available fossils of the family. The analysis was based on the combined *mat*K and *rbc*L gene sequences and a sampling following the latest angiosperm phylogeny APG III [116]. In addition to the sequences of the slipper orchids and their close relatives determined in the present study, the *mat*K and *rbc*L sequences of 165 taxa were downloaded from GenBank (see Supplementary Table S3), which represent 119 genera of Orchidaceae, 24 genera of non-orchid Asparagales, 5 genera of Commelinids, and 3 genera of Liliales as outgroups. The final data matrix comprised 200 taxa, which are many more than that sampled in previous studies. Divergence times were estimated using the nonparametric rate smoothing (NPRS) [117] and penalized-likelihood (PL) [118] implemented in the program r8s v1.71 [119], and Bayesian inference in BEAST v1.5.4 [120]. Four calibration points were used for age estimation, including Dominican amber (15–20 Mya) as a minimum age constraint for the Goodyerinae [40], macrofossils of *Dendrobium* (20–23 Mya) and *Earina* (20–23 Mya) [41] as the lower bound of the two genera following Gustafsson et al. [42], the age of the oldest known Asparagales (93–105 Mya) as the minimum age of the root of the tree, and the age of the oldest known fossil monocot as the maximum age at the root of the tree (110–120 Mya) [121] following the phylogenetic placement of Ramírez et al. [40]. In the r8s analysis, the oldest and youngest ages of the fossils were used separately. In the BEAST analysis, the age was estimated with the tree priors set as follows: i) age for the Goodyerinae (a monophyletic subtribe) as uniform distribution with a lower bound of 15 Mya and an upper bound of 120 Mya; ii) age for both *Dendrobium* and *Earina* as uniform distribution (lower bound: 20 Mya; upper bound: 120 Mya); iii) age for the root of the tree with a normal prior distribution as 106.5±8.21 Mya (95% CI: 93–120 Mya) [42]. The above 200-taxa analysis showed that the age estimates by NPRS and PL are very close, but are older than that by BEAST (see Results section). Considering that the use of multiple gene sequences could yield a more accurate time estimation when a constant diversification rate among lineages is violated [122], we conducted a further analysis for the slipper orchids (35 taxa, including outgroups) using combined six chloroplast genes (combined cpDNA, including *mat*K, *rbc*L, *rpo*C1, *rpo*C2, *ycf*1, and *ycf*2) with NPRS and PL methods. We did not use the BEAST estimate in this analysis due to its wide confidence interval. The crown ages of Cypripedioideae were set to 64±4 Mya (oldest age) and 58±4 Mya (youngest age), according to the result of PL analysis on the 200-taxa dataset. For the PL method, a cross-validation procedure was used to determine the most likely smoothing parameter. To calculate the standard errors, one hundred bootstrapped trees with fixed topology were generated with PAUP version 4.0b10. In the Bayesian analysis, divergence times were estimated with a log normal relaxed molecular clock using the Yule model of speciation. We ran 20,000,000 generations of Markov chain Monte Carlo (MCMC), and sampled every 2000 generations, with a burn-in of 1000 trees. The MCMC output analysis was conducted with TreeAnnotator v1.5.4, and the chronological phylogeny was displayed by FigTree v1.3.1.

The ancestral distribution of slipper orchids was reconstructed with S-DIVA 1.9 beta [123], [124], and Lagrange [125], [126]. S-DIVA complements DIVA, and considers the phylogenetic uncertainty in DIVA optimization. We used the randomly sampled 9000 post-burnin trees derived from the BEAST analysis for ancestral area reconstruction. In contrast, as a likelihood-based method under the dispersal-extinction-cladogenesis model, Lagrange enables the estimation of ancestral states, and calculates the probabilities of the most-likely areas at each node. Based on the present distribution of slipper orchids, we directly divided it into two geographical areas, Old World and New World. The biogeographical data were coded based directly on the distribution of the studied species, and the distribution of outgroups was excluded due to its wideness.

## Results

### Sequence characterization

Six chloroplast genes (*mat*K, *rbc*L, *rpo*C1, *rpo*C2, *ycf*1, and *ycf*2) were successfully amplified and directly sequenced for all samples except the cloning of *ycf*1 from *Vanilla planifolia*. The amplification of *acc*D failed in one of the outgroups, and thus this gene was excluded from further analysis. The PCR products of primers *trn*N/*trn*L had great length variation in slipper orchids, ranging from ∼1400 bp to ∼6000 bp, which, together with the amplification results of primers *ndh*FcF/*ndh*FaR, suggests that the *ndh*F gene has been completely lost in *Mexipedium* and the studied species of *Phragmipedium* (see Supplementary Table S4). Hence, this gene was also excluded from the phylogenetic analysis. The amplified *mat*K region includes the complete *mat*K coding sequence and ∼180 bp of the *trn*K intron. It is interesting that only a pseudogene of *mat*K, with a frameshift mutation and an early stop codon, was obtained from *Vanilla* sp. Although we tried to clone the PCR product and to amplify the gene with redesigned primers specific to *Vanilla*, the functional copy of *mat*K was still not found. Actually, several previous studies have reported that the functional *mat*K gene does not occur in some orchids [127]–[129]. The *mat*K pseudogene of *Vanilla* sp. was finally used in the phylogenetic analysis, since it only differs from the sequence of its congeneric species in several nucleotide substitutions and three nontriplet indels (5 bp insertion, 13 bp insertion, and 4 bp deletion). The amplification products of the *rpo*C1 gene cover about 1300 bp coding and about 800 bp intron sequences. The direct sequencing chromatogram of *ycf*1 from *Vanilla planifolia* showed double-peaks, and therefore we cloned the purified PCR product. Consequently, we obtained two distinct sequences of *ycf*1 from the species, both of which can be successfully translated. We chose the *ycf*1 copy that shows a higher similarity with the other outgroup species. A summary of the sequences that we used is shown in Table 2. Among the chloroplast genes, *ycf*1 is the most variable and parsimony-informative.

**Table 2:** Sequence information of the genes used in the present study.

Genes	Length (bp)		Alignment Length (bp)		Pi		Parsimony-informative sites	
	Within Cypripedioideae	Entire dataset	Within Cypripedioideae	Entire dataset	Within Cypripedioideae	Entire dataset	Within Cypripedioideae	Entire dataset
***mat*** **K**	1492–1518	1470–1523	1545	1581	0.04238	0.05872	175	302
***rbc*** **L**	1266	1266	1266	1266	0.01132	0.01679	42	90
***rpo*** **C1**	2003–2096	1962–2096	2056*	2090*	0.01959*	0.02892*	91*	210*
***rpo*** **C2**	2616–2661	2616–2691	2712	2811	0.02393	0.03884	159	415
***ycf*** **1**	1570–1690	1405–1690	1831	1870	0.05108	0.07255	200	357
***ycf*** **2**	1449–1512	1449–1644	1575	1836	0.00566	0.01809	17	157
**combined cpDNA**	10431–10627	10400–10627	10985*	11454*	0.02570*	0.03875*	684*	1531*
***ACO*** ** Exon**	780–795	780–795	798	801	0.08798	0.11352	204	303
***LFY*** ** Exon**	912–933	912–945	942	978	0.08745	0.11450	172	239
**combined nuclear DNA**	1704–1725	1704–1734	1740	1776	0.08965	0.11381	306	439
**Total**	12141–12323	12134–12375	12683*	13173*	0.03349*	0.04854*	915*	1736*

* The unalignable regions of the *rpo*C1 intron were excluded from our analyses.

The *LFY* gene of the slipper orchids amplified with primers *LFY*E1jF and *LFY*E3kR ranges from 1853 bp to 3717 bp in length, including partial sequences of exon 1 (258–270 bp) and exon 3 (234 bp), and complete sequences of exon2 (417–432 bp) and the two introns. In the three species *Phragmipedium besseae*, *Paphiopedilum delenatii* and *P. vietnamense*, the *LFY* gene amplified with primers *LFY*E1jF and *LFY*E2S5R includes partial sequence of exon1 and almost the whole length of exon2 and intron 1. Unfortunately, none of the two primer pairs worked in the five species of *Cypripedium* (*C. californicum*, *C. candidum*, *C. farreri*, *C. debile* and *C. palangshanense*). The intron sequences cannot be reliably aligned among the five genera of slipper orchids, and thus were excluded from our analyses. Except the failure of PCR amplification in *Phragmipedium besseae* and *Neuwiedia singapureana*, we got the *ACO* gene from all the other samples of slipper orchids, which ranges from 909 bp to 2178 bp in length. After excluding the introns, due to the difficulty in aligning, the coding region of *ACO* ranges from 780 bp to 795 bp in length (Table 2). The *ACO* gene has four exons and three introns except the loss of the second intron in the two genera *Mexipedium* and *Phragmipedium*, *Apostasia* sp. and two species of *Cypripedium* (*C. fasciculatum* and *C. palangshanense*), and the loss of the third intron in the two species of *Vanilla*.

### Phylogenetic analysis, molecular dating and ancestral area reconstruction

Since the plastid genome behaves as a single locus, we directly combined the six chloroplast genes into a single dataset (combined cpDNA) for phylogenetic analysis. The MP analysis generated 60 equally most parsimonious trees (MPTs), with tree length = 4620 steps, consistency index (CI) = 0.78, and rentention index (RI) = 0.84. The ML and Bayesian trees of the combined cpDNA are nearly identical to the MP trees in topology except the slight difference in interspecific relationships of *Cypripedium* and the weak bootstrap support for the position of *Selenipedium* in the MP trees. The ML tree is shown in Supplementary Fig. S1. The nuclear gene analyses generated 1807 MPTs for *ACO* (tree length = 859 steps, CI = 0.67, RI = 0.85), and 80 MPTs for *LFY* (tree length = 966 steps, CI = 0.70, RI = 0.86). Also, the MP trees of the nuclear genes are identical to the ML and Bayesian trees in topology except a minor difference in the *Cypripedium* clade (see ML trees in Supplementary Figs. S2, S3).

**Figure 2:**
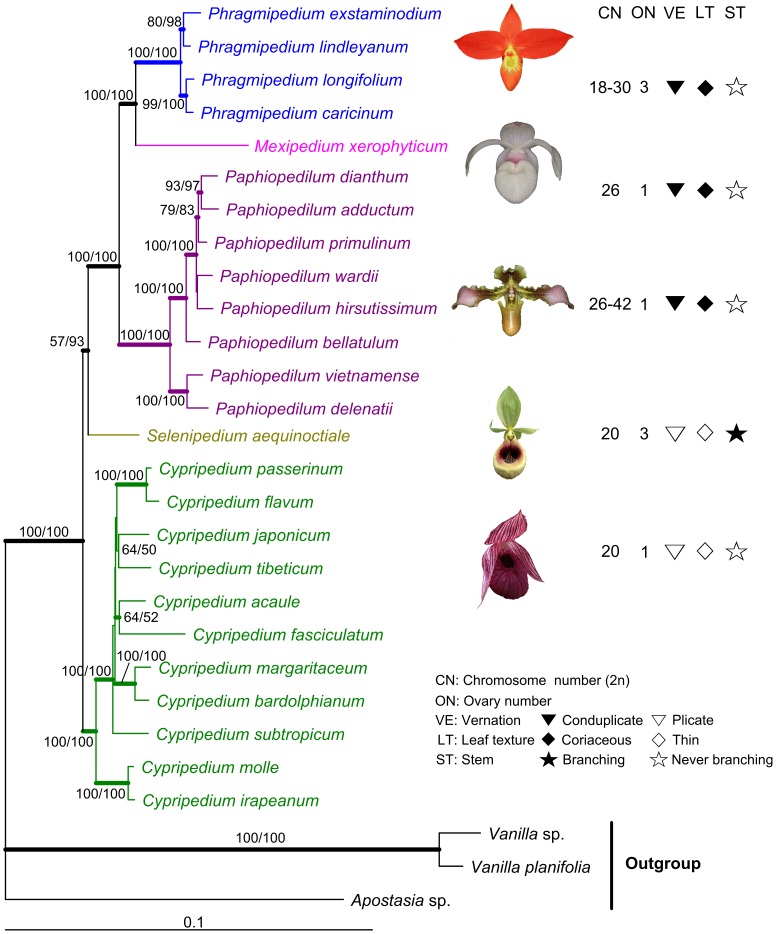
The ML tree of slipper orchids constructed based on the combined cpDNA+nuclear genes. Numbers above branches indicate the bootstrap values ≥50% for the MP and ML analyses, respectively. Bayesian posterior probabilities (≥0.90) are shown in bold lines. Symbols on the right indicate the distribution of some important characters of slipper orchids.

Since the ILD test did not detect significant incongruence between the two nuclear genes (p = 0.69) and between combined cpDNA and nuclear DNA (p = 0.50), we further conducted phylogenetic analyses using the two combined datasets. As a result, 27 MPTs were generated for the combined nuclear genes (tree length = 1621 steps, CI = 0.71, RI = 0.80), and 6 MPTs were generated for the combined cp- and nuclear DNA (tree length = 5284 steps, CI = 0.79, RI = 0.84), respectively. The ML and Bayesian trees generated based on the two combined datasets show the same intergeneric relationships of slipper orchids as in the MP trees (see ML trees in Supplementary Fig. S4; Fig. 2).

All gene trees generated in the present study, either based on separate genes or on combined datasets (Fig. 2; Supplementary Figs. S1, S2, S3, S4), are consistent about the intergeneric relationships of slipper orchids. That is, the widespread *Cypripedium* diverged first, followed by *Selenipedium* from South America, and finally the three conduplicate genera. The monotypic genus *Mexipedium* is most closely related to the South American *Phragmipedium*, and the two New World genera form a clade sister to the Old World *Paphiopedilum* (Fig. 2; Supplementary Figs. S1, S2, S3, S4).

**Figure 3:**
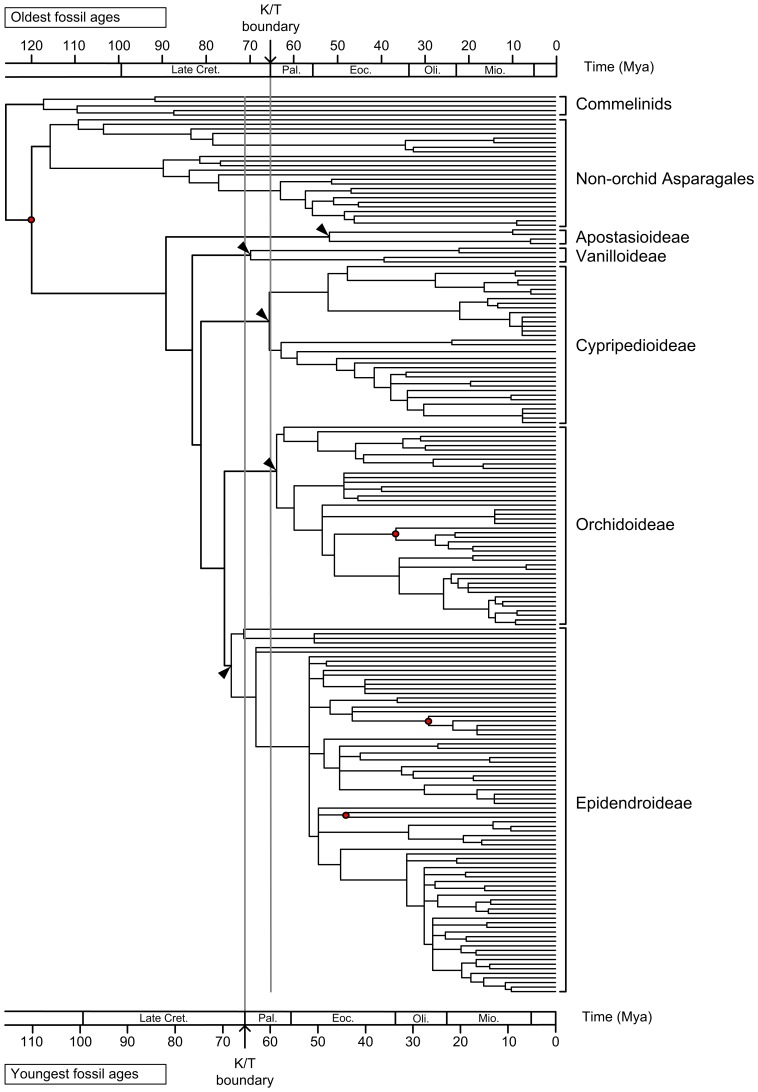
Fossil-calibrated molecular chronogram of the family Orchidaceae based on combined *mat*K+*rbc*L sequences. Red circles indicate age-constrained nodes, and arrows indicate the crown ages of the five subfamilies of Orchidaceae.

**Figure 4:**
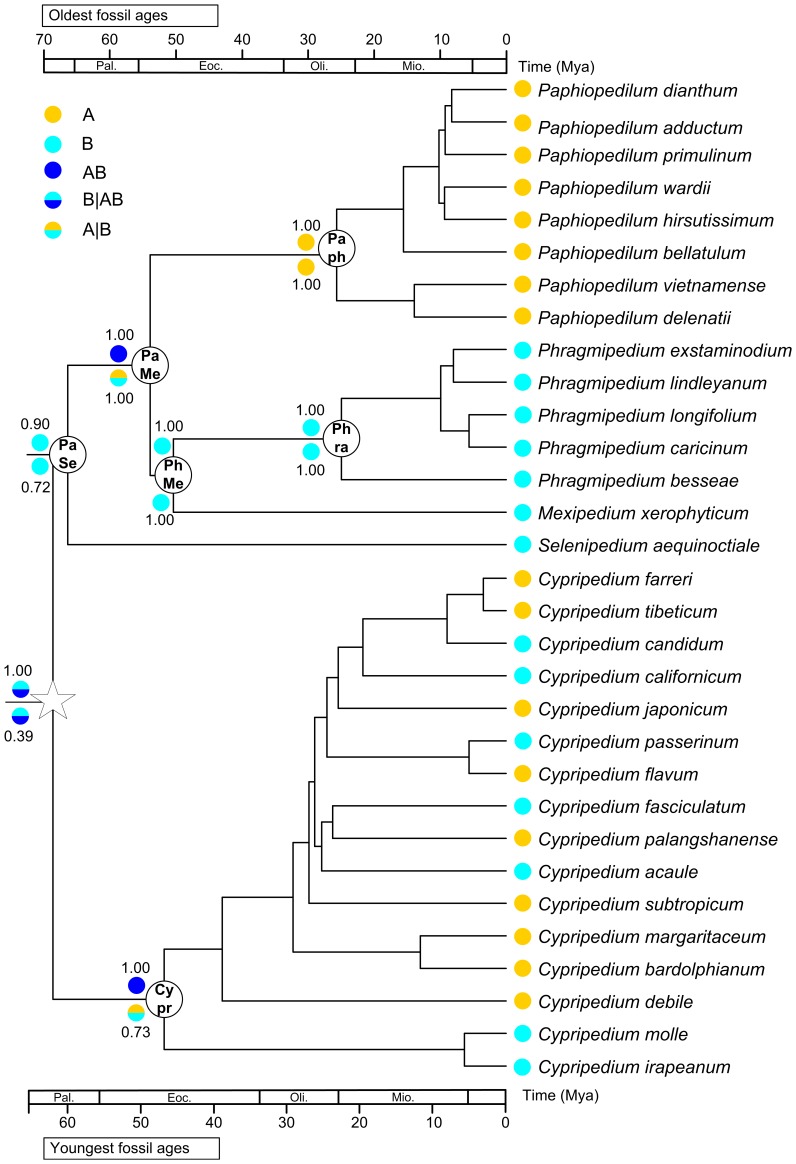
Chronogram of slipper orchids inferred from the combined six chloroplast genes, and ancestral area reconstruction. The crown age of slipper orchids was set as a calibration point for time estimation. Two areas were defined: (A) Old World and (B) New World. The ancestral areas with the highest probabilitiy are shown above (S-DIVA) and below (Lagrange) the branches with pie charts.

The LRT test rejected a clock-like evolution of combined *mat*K+*rbc*L (δ = 1815.1177, df = 198, P<0.001) and combined six chloroplast genes (δ = 5839.3257, df = 33, P<0.001). Therefore, we used NPRS and PL in r8s and Bayesian methods to estimate the divergence times. The family-level analysis (200 taxa) showed that the crown ages of Orchidaceae and its five subfamilies are older than the estimates by previous studies [40], [42], although the BEAST estimates showed a wide range (Table 3, Fig. 3). It is interesting that the crown ages of the subfamily Cypripedioideae estimated by NPRS and PL in the present study are very close, not as in Ramírez et al. [40] that obtained very different estimates by the two methods. This implys that a good sampling is important for molecular dating. The divergence times within Cypripedioideae estimated from the combined six chloroplast genes are generally congruent with those from the family-level analysis (Table 3). According to the age estimate, the genus *Selenipedium* originated in Palaeocene, while the most recent common ancestors of conduplicate slipper orchids (*Mexipedium*, *Phragmipedium* and *Paphiopedilum*) and of *Cypripedium* could be dated back to the Eocene (Table 3, Figs. 3, 4). Since the divergence times estimated with NPRS and PL are very close (Table 3), and thus only the PL estimates were used in the discussion. The ancestral area reconstruction suggests a New World origin or a wide ancestral distribution of slipper orchids, and indicates that vicariance is responsible for the disjunct distribution of conduplicate slipper orchids in palaeotropical and neotropical regions (Fig. 4).

**Table 3:** Estimated divergence times (Mya) derived from BEAST and r8s.

Node		*mat*K+*rbc*L				combined six chloroplast genes			
	BEAST	r8s				r8s			
	Median	Oldest ages		Youngest ages		Oldest ages		Youngest ages	
	(95% HPD)	NPRS	PL	NPRS	PL	NPRS	PL	NPRS	PL
**Family Orchidaceae**	87 (73–102)	88±3	89±2	81±2	82±2	—	—	—	—
**Subfam. Apostasioideae**	43 (25–64)	52±4	50±4	48±3	45±4	—	—	—	—
**Subfam. Vanilloideae**	66 (52–81)	74±3	74±3	67±3	68±3	—	—	—	—
**Subfam. Cypripedioideae**	43 (32–56)	64±4	64±4	59±4	58±4	—	—	—	—
**Cypr**	33 (23–45)	57±4	56±4	52±4	51±5	53.5±13.7	53.6±13.8	48.8±12.5	48.9±12.5
**PaSe**	—	—	—	—	—	60.4±15.4	60.4±15.4	55.1±14.0	55.1±14.0
**PaMe**	33 (23–43)	50±4	49±4	46±4	45±4	46.8±12.0	46.5±11.9	42.7±10.9	42.3±10.9
**PhMe**	27 (18–37)	44±4	43±4	41±4	39±4	43.1±11.1	42.7±11.0	39.3±10.1	38.9±10.0
**Paph**	18 (11–26)	28±4	27±4	26±4	25±4	24.2±6.4	22.2±5.9	22.1±5.9	20.0±5.4
**Phra**	15 (8–21)	26±4	25±4	24±4	23±4	25.9±7.0	24.4±6.7	23.6±6.4	22.1±6.1
**Subfam. Orchidoideae**	63 (51–75)	65±3	67±3	60±3	62±3	—	—	—	—
**Subfam. Epidendroideae**	55 (42–68)	73±3	74±3	67±3	68±3	—	—	—	—

## Discussion

### Phylogeny and evolution of the slipper orchids

In previously reported phylogenies of slipper orchids, the main discrepancies are phylogenetic positions of *Selenipedium* and *Mexipedium*. Atwood [62] proposed *Selenipedium* be merged into *Cypripedium*. The morphological study [61] and the combination analysis of morphological and *rbc*L data [63] as well as the nrDNA ITS tree [64] indicate that *Selenipedium* is basal to the other slipper orchids, whereas the phylogenies based on the low copy nuclear gene *Xdh* [48], *atp*B [130] and the combined *mat*K+*rbc*L [47] suggest a basal position of *Cypripedium*. On the other hand, nrDNA ITS [64] and cpDNA [43], [46] trees supports a sister relationship between the two North American genera *Mexipedium* and *Phragmipedium*, whereas the *Xdh* tree indicates that *Mexipedium* is most closely related to the Old World *Paphiopedilum* [48].

Like the unstable phylogenetic position, *Selenipedium* also has a very interesting morphology. This genus has fragrant and crustose seeds like *Vanilla*, but has the same chromosome number (2n = 20) [131], valvate sepal aestivation, and leaf vernation and texture as *Cypripedium* (Fig. 2), and even shares some anatomical features with *Cypripedium irapeanum* and *C. californicum*. In addition, the three-locular ovary and the multi-flower inflorescence with one flower opening at a time in *Selenipedium* seem to be primitive features [51], [62], [92]. Moreover, *Selenipedium* is similar to the conduplicate-leaved genera in having persistent perianth [62].

*Mexipedium* is a monotypic genus endemic to Oaxaca of Mexico. Albert and Chase [50] established this genus, to which the species initially published as *Phragmipedium xeropedium* was transferred [132]. Similar to the situation in *Selenipedium*, the genus *Mexipedium* not only shares characters with *Phragmipedium* (e.g. valvate sepal aestivation), but also with *Paphiopedilum* (e.g. unilocular ovary). Due to the limited markers used, the phylogenetic position of *Mexipedium* was not consistent among several previous molecular phylogenetic studies [43], [46], [48], [64].

The phylogenetic relationships among the genera of slipper orchids are relatively well resolved in the present study, given the topological consistency among the gene trees generated either from cpDNA or from the low copy nuclear genes (Fig. 2; Supplementary Figs. S1, S2, S3, S4). We found that *Cypripedium* diverged first, followed by *Selenipedium*, and finally the three conduplicate genera, although the sister relationship between *Selenipedium* and the conduplicate genera is not very strongly supported (Fig. 2). That is, the plicate-leaved genera could be more primitive, while the conduplicate-leaved genera are more advanced. We also found that the two New World genera *Mexipedium* and *Phragmipedium* are most closely related and form a clade sister to the Old World *Paphiopedilum* (Fig. 2; Supplementary Figs. S1, S2, S3, S4). Moreover, the close relationship between the two neotropical conduplicate genera is corroborated by the shared loss of the *ndh*F gene. Based on the combined chloroplast and nuclear gene phylogeny (Fig. 2), in slipper orchids, the coriaceous conduplicate leaf has a single origin, but ovary number is not phylogenetically informative.

### Biogeography of the slipper orchids: Implications for the evolution of Orchidaceae

The biogeographical history of slipper orchids is of great interest, but still remains controversial. Atwood [62] and Albert [63] put forward that slipper orchids were once widely distributed in North America/Asia, and that its current disjunct distribution was shaped by the separation of continents and the climatic cooling in the Ice Ages. Cox et al. [64] suggested southern North America/Mesoamerica as the origin center of slipper orchids based on the nrDNA ITS analysis. However, the reconstruction of biogeographical history should be based on a solid phylogeny, divergence time estimation and ancestral area reconstruction. In the relatively well-resolved phylogeny of slipper orchids reconstructed in the present study, *Cypripedium*, a genus with a wide distribution in temperate and subtropical North Hemisphere, is basal to the other genera. Also, the PL estimate suggests a Palaeocene origin of *Selenipedium*, while the most recent common ancestors of conduplicate slipper orchids and of *Cypripedium* could be dated back to the Eocene (Table 3, Figs. 3, 4). Although no available fossils of slipper orchids can be used for time calibration, the time estimates from combined *mat*K+*rbc*L using other orchid fossils as calibration points are generally congruent with those from the combined six chloroplast genes using a secondary calibration point. It is well known that the climatic cooling or oscillation since Eocene/Oligocene [133], [134] has led to great changes in plant distribution patterns. Therefore, although southern North America/Mesoamerica has three out of the five genera (*Cypripedium*, *Phragmipedium* and *Mexipedium*) of slipper orchids, this region is very likely a museum rather than a cradle for the diversity. In fact, *Phragmipedium* is mainly distributed in South America. The ancestral area reconstruction also suggests that the common ancestor of slipper orchids occurred in the New World or had a wide distribution in both Old and New Worlds (Fig. 4).

The Isthmus of Panama had served as a corridor for flora and fauna exchange between North America and South America before 3–3.5 Mya, which may explain the distribution of *Selenipedium* and *Phragmipedium* in South America. For instance, pollen records and vertebrate fossils from the Caribbean region indicate that the GAARlandia land bridge had connected North and South America during Eocene-Oligocene (35-33 Mya) [135]. In addition, Iturralde-Vinent & MacPhee [135] and Pennington & Dick [136] both suggested the existence of a land bridge between the two continents in Miocene. Furthermore, the study of the palm tribe Chamaedoreeae also supports the Middle Eocene and Miocene migrations of plants between North and South America [137].

It is very interesting that the Old World *Paphiopedilum* is sister to a clade comprising the two New World genera *Mexipedium* and *Phragmipedium* (Fig. 2; Supplementary Figs. S1, S4), suggesting a vicariant differentiation of the conduplicate genera between the Old World and New World tropics. The three conduplicate genera occur in both the Northern and Southern Hemispheres, also including South America and a part of Southeast Asia from the Gondwanaland [138], [139]. According to many previous studies on other plant groups, the neotropical and palaeotropical disjunction could be explained by: (1) Gondwana breakup [140], [141], (2) trans-Pacific long distance dispersal [142], [143], and (3) fragmentation of the boreotropical flora [37], [66]. However, the first two hypotheses are not suitable for the conduplicate slipper orchids, although they can not be completely ruled out.

First, the crown age of slipper orchids was dated back to Palaeocene (Table 3; Fig 3), which is much younger than the time of Gondwana breakup, and slipper orchids do not occur in Australia and Africa. Therefore, the present distribution pattern of slipper orchids cannot be attributed to the Gondwana breakup. Second, trans-Pacific long distance dispersal is not supported by the reciprocal monophyly of the conduplicate slipper orchids from both sides of the Pacific Ocean, particularly the monophyly of the New World conduplicate slipper orchids comprising the two genera *Mexipedium* and *Phragmipedium*, and not by the divergence time estimation. That is, the conduplicate genera have a crown age of 42.3±10.9 Mya (youngest age) to 46.5±11.9 Mya (oldest age, in the Eocene), but the most recent common ancestors of *Paphiopedilum* and *Phragmipedium* are dated back to 22.2±5.9 Mya (oldest age) and 24.4±6.7 (oldest age) Mya, respectively (Table 3, Fig. 4). The estimated divergence times suggest an early origin for each of the conduplicate genera but a much later diversification or the extinction of ancient species within the genera. It is very likely that vicariant differentiation is responsible for the disjunct distribution of the conduplicate genera between the Old World and New World tropics. That is, the ancestor of the conduplicate slipper orchids could have a continuous distribution in the boreotropics, and migrated southwards to both sides of the Pacific Ocean due to the climate cooling in the late Cenozoic [23], [134], and then evolved into separate genera. Although the seeds of orchids are tiny [144], which may facilitate long distance dispersal, Moles et al. [145] found that seed size is more associated with growth form than with dispersal syndrome. In fact, boreotropical vicariance was also reported in *Persea* [146] and *Parthenocissus* [147]. Additionally, the existence of a boreotropical flora is supported by many other plant biogeographic studies, such as in Burmanniaceae [18], Chamaedoreeae [137], Rubiaceae [148], and Annonaceae [149]. The high latitude of the Bering land bridge made it a barrier for the migration of thermophilic plants but still a corridor for the exchange of temperate plants like *Cypripedium*. According to the divergence times and distributions of different lineages of *Cypripedium*, multiple events of vicariance and dispersal between East Asia and North America could have occurred in the genus from middle to late Tertiary (Fig. 4).

The phylogenetic and biogeographic history of slipper orchids revealed in the present study may shed some lights on the evolution of Orchidaceae, one of the largest families of angiosperms with ∼850 genera and ∼25,000 species recorded [39]. A series of studies have investigated the mechanisms underlying the high diversity of orchids, such as epiphytism and pollinator specialization [150], deceptive pollination [151], mycorrhizal fungi [152], crassulacean acid metabolism [153], and reduction of evolutionary constraints on the class B floral homeotic genes [154]. However, the previous studies mainly focused on the key characters of orchids, and paid little attention to the impacts of climatic oscillations and geological events, which are important driving forces of speciation [155]–[157].

In *Cypripedium*, the basal clade of slipper orchids (Fig. 2; Supplementary Figs. S1, S4), the most ancestral species are distributed in subtropical Mexico (Fig. 2; Supplementary Fig. S1), although most species of the genus are confined to the temperate Northern Hemisphere. Interestingly, the basal species of *Paphiopedilum*, a mainly tropical genus, also occur in the subtropics (southwest China and Vietnam) (Fig. 2; Supplementary Figs. S1, S4). That is, although the largest two genera of slipper orchids (*Cypripedium* and *Paphiopedilum*) have very different distributions, both of them seem to have an origin in the subtropics. This may suggest that their high species diversity and present wide distribution, either in temperate or in tropical regions, were developed to adapt to new niches created by climatic oscillations in the late Cenozoic. Actually, according to anatomical structures, plicate (*Cypripedium*) and conduplicate (*Paphiopedilum*) leaves can really adapt to different environments [158].

Moreover, previous biogeographical studies of orchids mainly focused on some endemic genera, e.g. *Bromheadia* and *Holcoglossum* in Southeast Asia [159], [160], *Antilles* in the neotropics [161], and *Caladenia* in Australia [162], except a couple of them that dealt with widely distributed genera, e.g. *Vanilla* [163] and *Polystachya* [164]. In the present study, we sampled all five genera of slipper orchids, including both endemic and widespread ones, and found the vicariant differentiation of the conduplicate genera between the Old World and New World tropics. Obviously, to interpret the nearly cosmopolitan distribution of Orchidaceae (except poles and deserts) [39], the future biogeographical study of orchids should include both regional endemics and their widespread relatives, which will be also helpful to achieve a widely-accepted classification of orchids, particularly at the genus level.

## Supporting Information

Figure S1 **The ML tree of the slipper orchids constructed based on the combined six chloroplast genes.** Numbers above branches indicate bootstrap values ≥50% for the MP and ML analyses, respectively. Bayesian posterior probabilities (≥0.90) are shown in bold lines.
(TIF)

Figure S2 **The ML tree of the slipper orchids constructed based on the nuclear** ***ACO*** **gene.** Numbers above branches indicate bootstrap values ≥50% for the MP and ML analyses, respectively. Bayesian posterior probabilities (≥0.90) are shown in bold lines. Numbers following the species names are the clone numbers.
(TIF)

Figure S3 **The ML tree of the slipper orchids constructed based on the nuclear** ***LFY*** **gene.** Numbers above branches indicate bootstrap values ≥50% for the MP and ML analyses, respectively. Bayesian posterior probabilities (≥0.90) are shown in bold lines. Numbers following the species names are the clone numbers.
(TIF)

Figure S4 **The ML tree of the slipper orchids constructed based on the combined nuclear genes.** Numbers above branches indicate bootstrap values ≥50% for the MP and ML analyses, respectively. Bayesian posterior probabilities (≥0.90) are shown in bold lines.
(TIF)

Table S1 **Sources of materials.**
(DOC)

Table S2 **PCR (P) and sequencing (S) primers used in this study.**
(DOC)

Table S3 **GenBank accession numbers of taxa used in this study.**
(DOC)

Table S4 **Amplification results of the** ***ndh*** **F gene with different primer pairs in the present study.**
(DOC)
