# Supplementary figures and images for: Evolution and Biogeography of the Slipper Orchids: Eocene Vicariance of the Conduplicate Genera in the Old and New World Tropics
Source: PLoS One. 2012 Jun 7;7(6):e38788. doi: 10.1371/journal.pone.0038788 (PMC3369861; doi:10.1371/journal.pone.0038788)

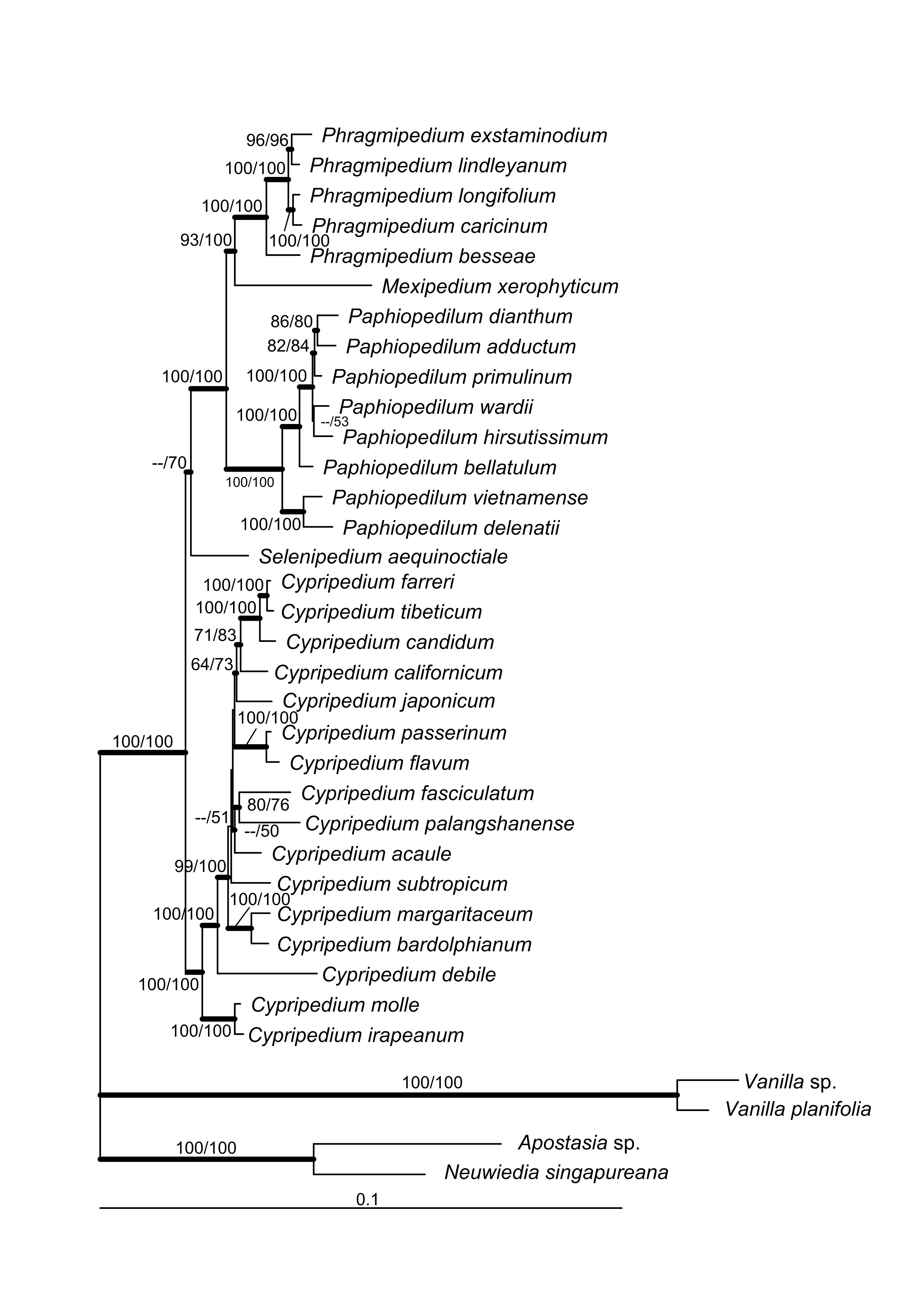

Supplement: Figure S1 — The ML tree of the slipper orchids constructed based on the combined six chloroplast genes. Numbers above branches indicate bootstrap values ≥50% for the MP and ML analyses, respectively. Bayesian posterior probabilities (≥0.90) are shown in bold lines. (TIF) [file pone.0038788.s001.tif]

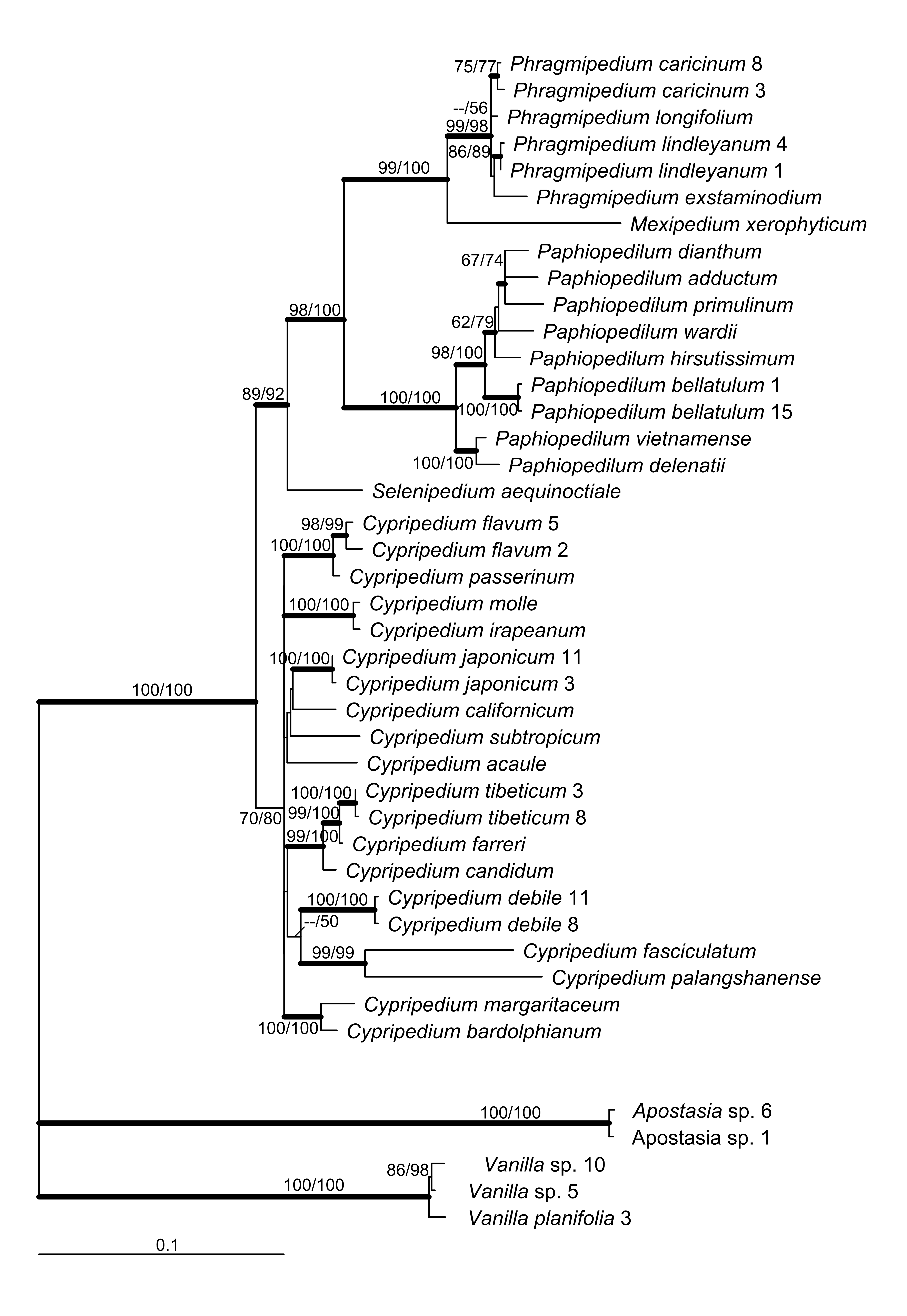

Supplement: Figure S2 — The ML tree of the slipper orchids constructed based on the nuclear ACO gene. Numbers above branches indicate bootstrap values ≥50% for the MP and ML analyses, respectively. Bayesian posterior probabilities (≥0.90) are shown in bold lines. Numbers following the species names are the clone numbers. (TIF) [file pone.0038788.s002.tif]

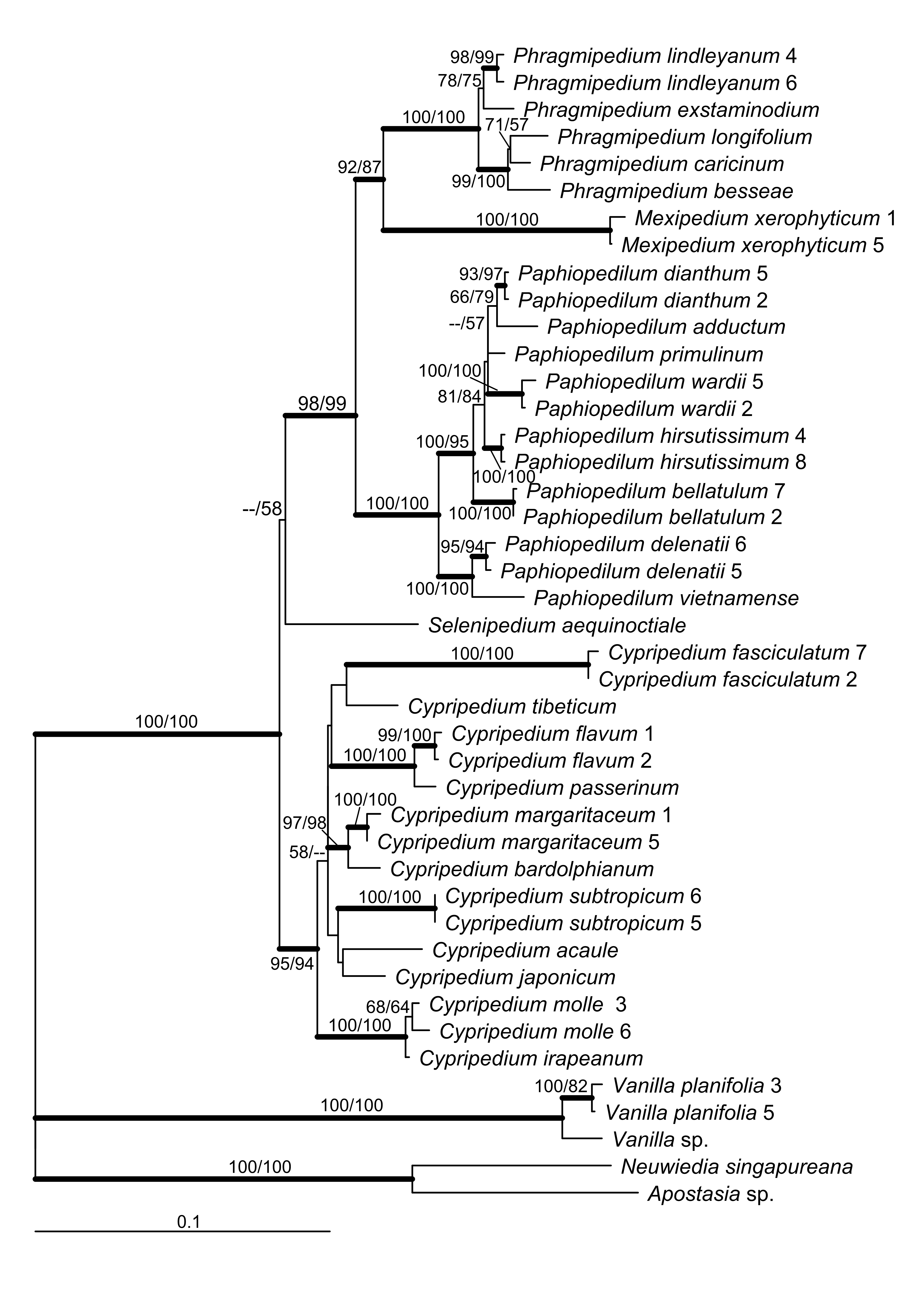

Supplement: Figure S3 — The ML tree of the slipper orchids constructed based on the nuclear LFY gene. Numbers above branches indicate bootstrap values ≥50% for the MP and ML analyses, respectively. Bayesian posterior probabilities (≥0.90) are shown in bold lines. Numbers following the species names are the clone numbers. (TIF) [file pone.0038788.s003.tif]

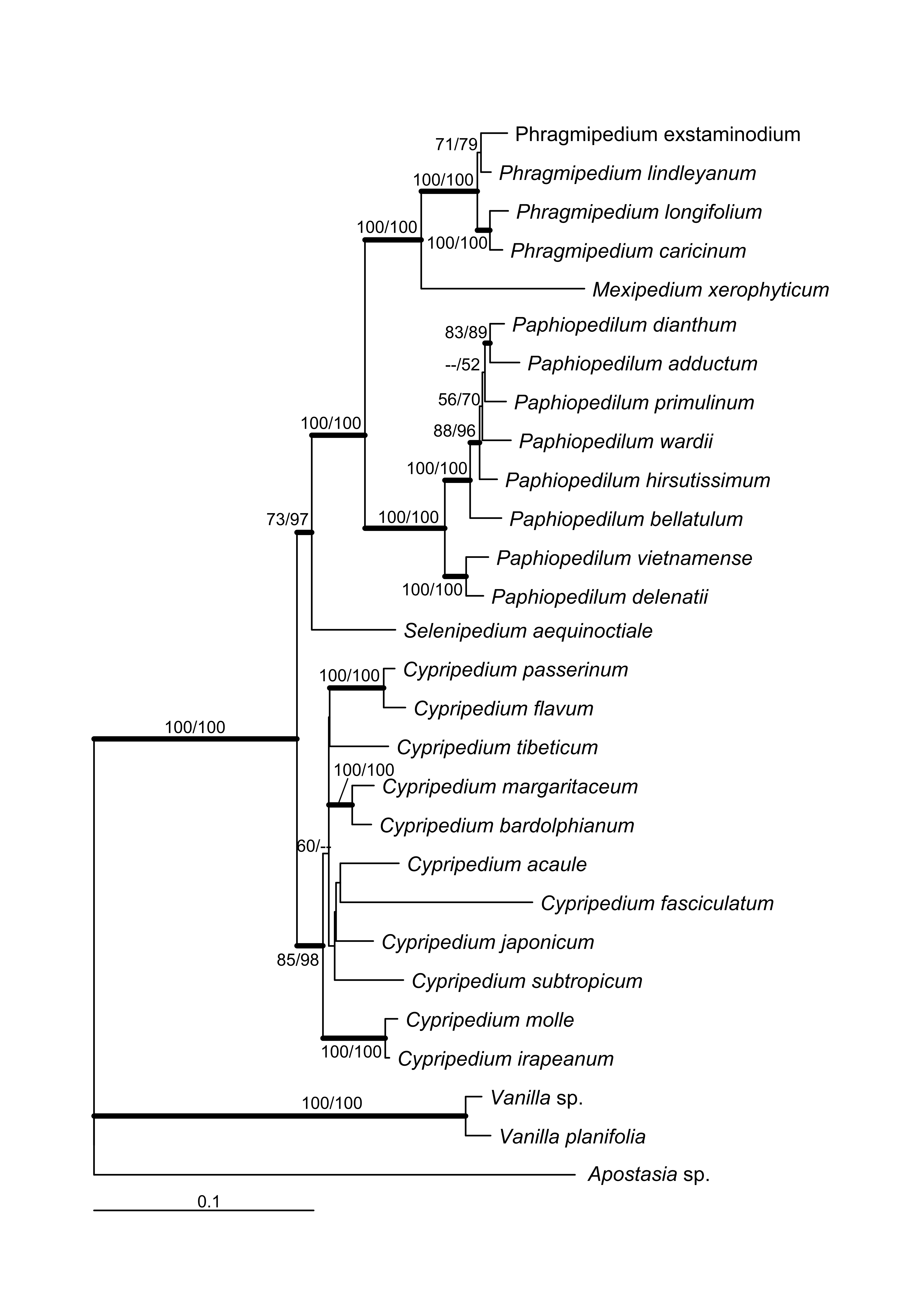

Supplement: Figure S4 — The ML tree of the slipper orchids constructed based on the combined nuclear genes. Numbers above branches indicate bootstrap values ≥50% for the MP and ML analyses, respectively. Bayesian posterior probabilities (≥0.90) are shown in bold lines. (TIF) [file pone.0038788.s004.tif]
