# Supplementary material for: Evolution and Biogeography of the Slipper Orchids: Eocene Vicariance of the Conduplicate Genera in the Old and New World Tropics
Source: PLoS One. 2012 Jun 7;7(6):e38788. doi: 10.1371/journal.pone.0038788 (PMC3369861; doi:10.1371/journal.pone.0038788)
Supplement: Table S1 — Sources of materials. (DOC) [file pone.0038788.s005.doc]

**Table S1. Sources of materials.**

|  | **Taxon** | **Sources/Vouchers** | **GenBank accession numbers** | | | | | | | |
| --- | --- | --- | --- | --- | --- | --- | --- | --- | --- | --- |
|  |  |  | ***mat*K** | ***rbc*L** | ***rpoC*1** | ***rpoC*2** | ***ycf*1** | ***ycf*2** | ***ACO*** | ***LFY*** |
| ***Cypripedium*** |  |  |  |  |  |  |  |  |  |  |
| Sect. *Subtropica* | *C.* *subtropicum* S.C.Chen & K.Y.Lang | Tibet, China/Jin X.-H., N8003(PE) | JQ182206 | JQ182224 | JQ182242 | JQ182260 | JQ182278 | JQ182296 | JQ182168 | JQ182182  JQ182183 |
| Sect. *Irapeana* | *C. californicum* A.Gray | USA (Cult.)/ Gunnlaugson D. S. 1144 | JQ182201 | JQ182219 | JQ182237 | JQ182255 | JQ182273 | JQ182291 | JQ182158 | - |
|  | *C. irapeanum* Lex. in P.de La Llave & J.M.de Lexarza | Mexico/Salazar G. A. 7234 | JQ182207 | JQ182225 | JQ182243 | JQ182261 | JQ182279 | JQ182297 | JQ182169 | JQ182184 |
|  | *C. molle* Lindl. in G.Bentham | Mexico/Salazar G. A. 6883 | JQ182208 | JQ182226 | JQ182244 | JQ182262 | JQ182280 | JQ182298 | JQ182170 | JQ182185  JQ182186 |
| Sect. *Obtusipetala* | *C. flavum* P.F.Hunt & Summerh | Lijiang, China/Yang F.-S., ZWY 23 | JN181457 | JN181474 | JN181491 | JN181508 | JN181525 | JN181542 | JN181415  JN181416 | JN181438  JN181439 |
|  | *C. passerinum* Richardson | Canada/Kevan P. S. | JQ182200 | JQ182218 | JQ182236 | JQ182254 | JQ182272 | JQ182290 | JQ182167 | JQ182180 |
| Sect. *Cypripedium* | *C. candidum* Muhl. ex Willd. | USA(Cult.)/Steele B. S. 1299 | JQ182199 | JQ182217 | JQ182235 | JQ182253 | JQ182271 | JQ182289 | JQ182164 | - |
|  | *C. farreri* W.W.Sm. | Sichuan, China/Li J.-H. S6181(NOCC) | JQ182198 | JQ182216 | JQ182234 | JQ182252 | JQ182270 | JQ182288 | JQ182163 | - |
|  | *C. tibeticum* King ex Rolfe | Sichuan, China/Li J.-H. S6132(NOCC) | JQ182197 | JQ182215 | JQ182233 | JQ182251 | JQ182269 | JQ182287 | JQ182161  JQ182162 | JQ182177 |
| Sect. *Enantiopedilum* | *C. fasciculatum* Kellogg | USA/Ross Kouzes, N6160(PE) | JN181460 | JN181477 | JN181494 | JN181511 | JN181528 | JN181545 | JN181420 | JN181442  JN181443 |
| Sect. *Flabellinervia* | *C. japonicum* Thunb. in J.A.Murray | Hubei, China/Li J.-H., E6701(NOCC) | JN181458 | JN181475 | JN181492 | JN181509 | JN181526 | JN181543 | JN181417  JN181418 | JN181440 |
| Sect. *Acaulia* | *C. acaule* Aiton | USA/Gill D. E. | JQ182204 | JQ182222 | JQ182240 | JQ182258 | JQ182276 | JQ182294 | JQ182159 | JQ182181 |
| Sect. *Retinervia* | *C. debile* Rchb.f. | Sichuan, China/ Li J.-H, N6160 | JQ182205 | JQ182223 | JQ182241 | JQ182259 | JQ182277 | JQ182295 | JQ182165  JQ182166 | - |
|  | *C. palangshanense* Tang & F.T.Wang | Sichuan, China/Li J.-H, S6152(NOCC) | JQ182203 | JQ182221 | JQ182239 | JQ182257 | JQ182275 | JQ182293 | JQ182157 | - |
| Sect. *Trigonopedia* | *C. bardolphianum* W.W.Sm. & Farrer | Sichuan, China/Yang F.-S., W01 | JN181459 | JN181476 | JN181493 | JN181510 | JN181527 | JN181544 | JN181419 | JN181441 |
|  | *C. margaritaceum* Franch*.* | Yunnan, China/Yang F.-S | JQ182202 | JQ182220 | JQ182238 | JQ182256 | JQ182274 | JQ182292 | JQ182160 | JQ182178  JQ182179 |
| ***Mexipedium*** | *M. xerophyticum* (Soto Arenas, Salazar & Hágsater) V.A.Albert & M.W.Chase | Missouri Botanical Garden/902725-5 | JN181455 | JN181472 | JN181489 | JN181506 | JN181523 | JN181540 | JN181412 | JN181435  JN181436 |
| ***Paphiopedilum*** |  |  |  |  |  |  |  |  |  |  |
| Sect. *Parvisepalum* | *P. delenatii* Guillaumin | NOCC, China/1951 | JQ182193 | JQ182211 | JQ182229 | JQ182247 | JQ182265 | JQ182283 | JQ182154 | JQ182187  JQ182188 |
|  | *P. vietnamense* O.Gruss & Perner | NOCC, China/2090 | JQ182194 | JQ182212 | JQ182230 | JQ182248 | JQ182266 | JQ182284 | JQ182155 | JQ182189 |
| Sect. *Concoloria* | *P. bellatulum* (Rchb.f.) Stein | NOCC, China | JN181448 | JN181465 | JN181482 | JN181499 | JN181516 | JN181533 | JN181400  JN181401 | JN181424  JN181425 |
| Sect. *Coryopedilum* | *P. adductum* Asher | NOCC, China/2297 | JQ182191 | JQ182209 | JQ182227 | JQ182245 | JQ182263 | JQ182281 | JQ182152 | JQ182173 |
| Sect. *Pardalopetalum* | *P. dianthum* Tang & F.T.Wang | NOCC, China/2104 | JQ182192 | JQ182210 | JQ182228 | JQ182246 | JQ182264 | JQ182282 | JQ182153 | JQ182174  JQ182175 |
| Sect. *Cochlopetalum* | *P. primulinum* M.W.Wood & P.Taylor | Missouri Botanical Garden/882387-2 | JN181451 | JN181468 | JN181485 | JN181502 | JN181519 | JN181536 | JN181405 | JN181430 |
| Sect. *Paphiopedilum* | *P. hirsutissimum* (Lindl. Ex Hook.) Stein | NOCC, China/1784 | JN181449 | JN181466 | JN181483 | JN181500 | JN181517 | JN181534 | JN181402 | JN181426  JN181427 |
| Sect. *Barbata* | *P. wardii* Summerh. | NOCC, China/2701 | JN181450 | JN181467 | JN181484 | JN181501 | JN181518 | JN181535 | JN181404 | JN181428  JN181429 |
| ***Phragmipedium*** |  |  |  |  |  |  |  |  |  |  |
| Sect. *Micropetalum* | *P. besseae* Dodson & J.Kuhn | Missouri Botanical Garden/992661-1 | JQ182196 | JQ182214 | JQ182232 | JQ182250 | JQ182268 | JQ182286 | - | JQ182190 |
| Sect. *Phragmipedium* | *P. caricinum* (Lindl. & Paxton) Rolfe | Missouri Botanical Garden/951918-3 | JN181452 | JN181469 | JN181486 | JN181503 | JN181520 | JN181537 | JN181406  JN181407 | JN181431 |
|  | *P. exstaminodium* Castaño | Mexico/Salazar G. A. 12985 | JQ182195 | JQ182213 | JQ182231 | JQ182249 | JQ182267 | JQ182285 | JQ182156 | JQ182176 |
| Sect. *Lorifolia* | *P. longifolium* (Warsz. & Rchb.f.) Rolfe | Missouri Botanical Garden/840941-1 | JN181454 | JN181471 | JN181488 | JN181505 | JN181522 | JN181539 | JN181410 | JN181434 |
| Sect. *Platypetalum* | *P. lindleyanum* (M.R.Schomb. ex Lindl.) Rolfe | Missouri Botanical Garden/942583-8 | JN181453 | JN181470 | JN181487 | JN181504 | JN181521 | JN181538 | JN181408  JN181409 | JN181432  JN181433 |
| ***Selenipedium*** | *S. aequinoctiale* Garay | Poland, Marcin Górniak | JN181456 | JN181473 | JN181490 | JN181507 | JN181524 | JN181541 | JN181414 | JN181437 |
| **Outgroups** | *Vanilla planifolia* Jacks. Ex Andrews | Royal Botanical Garden, Kew/0-170 | JN181462 | JN181479 | JN181496 | JN181513 | JN181530 | JN181547 | JN181423 | JN181445 |
|  | *V.* sp. | Beijing Botanical Garden | JN181461 | JN181478 | JN181495 | JN181512 | JN181529 | JN181546 | JN181421  JN181422 | JN181444 |
|  | *Apostasia* sp. | NOCC, China | JN181464 | JN181481 | JN181498 | JN181515 | JN181532 | JN181549 | JQ182171  JQ182172 | JN181446 |
|  | *Neuwiedia singapureana* (Wall. Ex Baker) | NOCC, China | JN181463 | JN181480 | JN181497 | JN181514 | JN181531 | JN181548 | - | JN181447 |

NOCC: The National Orchid Conservation Center (The Orchid Conservation & Research Center of Shenzhen). Dashes indicate unavailable sequences.
