# Supplementary material for: Evolution and Biogeography of the Slipper Orchids: Eocene Vicariance of the Conduplicate Genera in the Old and New World Tropics
Source: PLoS One. 2012 Jun 7;7(6):e38788. doi: 10.1371/journal.pone.0038788 (PMC3369861; doi:10.1371/journal.pone.0038788)
Supplement: Table S2 — PCR (P) and sequencing (S) primers used in this study. (DOC) [file pone.0038788.s006.doc]

**Table S2. PCR (P) and sequencing (S) primers used in this study.**

| **Genes** | **Primers** |  | **Primer sequences（5’-3’）** | **Sources** |
| --- | --- | --- | --- | --- |
| ***mat*K** | *mat*K19F | P/S | CGTTCTGACCATATTGCACTATG | [1,2] |
|  | *mat*KR1 | P/S | CATTTTTCATTGCACACGRC | [3] |
|  | 390F | S | CGATCTATTCATTCAATATTTC | [4] |
|  | 1326R | S | TCTAGCACACGAAAGTCGAAGT | [4] |
|  | *mat*K559R | S | GAACCAAGATTTCCAGATG | this study |
|  | *mat*K1249F | S | CATCCTATTAGTAAACCGA | this study |
| ***rbc*L** | *rbc*LaF | P/S | GTTGTAGGGAGGGACTTATGT | this study |
|  | *rbc*LaR | P/S | TAGTTCAGGGCTCCATTTG | this study |
| ***rpo*C1** | *rpo*C1aF | P/S | TGGAGARATAGTTGGAGAGGT | this study |
|  | *rpo*C1aR | P/S | GTGAACAGCCATTTGATCC | this study |
|  | *rpo*C1SaF | S | CCTAGTATATTGCGATGTGTG | this study |
|  | *rpo*C1SaR | S | TCCTGACACATTACTAATTCC | this study |
| ***rpo*C2** | *rpo*C2aF | P/S | GGAACCDGGRACKCAACTA | this study |
|  | *rpo*C2aR | P/S | CGACCYCGTAGAGCAGCTT | this study |
|  | *rpo*C2SaF | S | CACAAGGATCAAGATCAAAT | this study |
|  | *rpo*C2SaR | S | ACTTTTGGAAGACCTTGCG | this study |
|  | *rpo*C2SbR | S | GATACTCAATCAAATCCTCTT | this study |
|  | r*po*C2ScR | S | CTTATTTCGATTCAACAAC | this study |
| ***ycf*1** | *ycf*1_3720F | P/S | TACGTATGTAATGAACGAATGG | [5] |
|  | *ycf*1_5500R | P/S | GCTGTTATTGGCATCAAACCAATAGCG | [5] |
|  | y*cf*1bF | P/S | GGAAAAACTGGTTAAAAGGTC | this study |
|  | *ycf*1bR | P/S | CCAATAGCGATTCATACAAGC | this study |
|  | *ycf*1SaF | S | ATCTGGACCAATGCACATATT | [5] (modified) |
|  | *ycf*1SaR | S | TTTAATTGGAATGATCCAAGG | [5] (modified) |
|  | y*cf*1SbF | S | TCTCTGATACCGCATCAA | this study |
|  | *ycf*1SbR | S | CGTCCCTTCTTATAGATAG | this study |
| ***ycf*2** | *ycf*2aF | P/S | TGAGTTACAGACAGAGTTAGA | this study |
|  | *ycf*2aR | P/S | ACAGCCCTTCCTATCTGATAG | this study |
| ***ndh*F** | *ndh*FcF | P/S | GCTTCAATGTTGGGATTAG | this study |
|  | *ndh*FaR | P/S | TTGGGAAGACTGAAAGAAGAG | this study |
| ***trn*N-*trn*L** | *trn*NbF(guu) | P/S | AACAGCCRACCGCTCTACCAC | this study |
|  | *trn*LaR(uag) | P/S | AAGAGCAGCGTGTCTACCAAT | this study |
| **ACO** | *ACO*E1aF | P | GCNTGYGAGAACTGGGGHTTCTTYGAG | this study |
|  | *ACO*E2aR | P | ATGGTCTTCATGGCCTCAAACCT | this study |
|  | *ACO*E1SaF | S | ACAACCTCGACTGGGAGAGC | this study |
|  | *ACO*E3SbR | S | GATGTTGACGACGATGGAGTG | this study |
| ***LFY*** | *LFY*E1jF | P | TGAGGGAGGAGGAGGTSGACGAYATGAT | this study |
|  | *LFY*E3kR | P | AGATBGAGAGGCGSGGATGSGCGTTGAA | this study |
|  | *LFY*E2S1F | S | AGGACTGTCAGARGAGCCKGTGC | this study |
|  | *LFY*E3S1R | S | TTCTTCGCGTACCGGAACACCAG | this study |
|  | *LFY*E2S2R | S | CGGCTCCTCTGACAGTCCTGC | this study |
|  | *LFY*E2S3F | S | TTCAGGTTCAGGCCGTCGCAAAG | this study |
|  | *LFY*E2S5R | P/S | AGGAGGAACTCATGGCACTG | this study |
|  | *LFY*E2S6F | S | CAGTGCCATGAGTTCCTC | this study |
|  | *LFY*I2S3R | S | CGTGTCTTACCATCTTCTATC | this study |
|  | *LFY*AE3SaR | S | GTATCTGAACACCTGGTTCGT | this study |
|  | *LFY*AI2SaF | S | GCACCAAACGCTCATAAGTCG | this study |

**References**

1. Kores PJ, Weston PH, Molvray M, Chase MW (2000) Phylogenetic relationships within the Diurideae (Orchidaceae): inferences from plastid *mat*K DNA sequences. In: Wilson KL, Morrison DA, editors. Monocots: Systematics and Evolution. Collingwood: CSIRO publishing. pp. 449-456.

2. Molvray M, Kores PJ, Chase MW (2000) Polyphyly of mycoheterotrophic orchids and functional influences on floral and molecular characters. In: Wilson KL, Morrison DA, editors. Monocots: Systematics and Evolution. Collingwood: CSIRO publishing. pp. 441-448.

3. Kocyan A, Qiu YL, Endress PK, Conti E (2004) A phylogenetic analysis of Apostasioideae (Orchidaceae) based on ITS, *trn*L-F and *mat*K sequences. Plant Syst Evol 247: 203-213.

4. Lahaye R, Van der Bank M, Bogarin D, Warner J, Pupulin F, et al. (2008) DNA barcoding the floras of biodiversity hotspots. Proc Natl Acad Sci USA 105: 2923-2928.

5. Neubig KM, Whitten WM, Carlsward BS, Blanco MA, Endara L, et al. (2009) Phylogenetic utility of *ycf*1 in orchids: a plastid gene more variable than *mat*K. Plant Syst Evol 277: 75-84.
