# Supplementary material for: Evolution and Biogeography of the Slipper Orchids: Eocene Vicariance of the Conduplicate Genera in the Old and New World Tropics
Source: PLoS One. 2012 Jun 7;7(6):e38788. doi: 10.1371/journal.pone.0038788 (PMC3369861; doi:10.1371/journal.pone.0038788)
Supplement: Table S3 — GenBank accession numbers of taxa used in this study. (DOC) [file pone.0038788.s007.doc]

**Table S4.** GenBank accession numbers of taxa used in this study.

|  |  | **Species** | ***mat*K** | ***rbc*L** |
| --- | --- | --- | --- | --- |
| Liliales | Colchicaceae | *Disporum sessile* | AB040182 | D17376 |
|  | Liliaceae | *Lilium superbum* | HQ180873 | HQ182436 |
|  | Melanthiaceae | *Trillium grandiflorum* | AB017392 | AY465712 |
| Zingiberales | Musaceae | *Musa acuminata* | EU016987 | AF378770 |
| Poales | Poaceae | *Oryza rufipogon* | JN005833 | JN005833 |
|  | Bromeliaceae | *Vriesea psittacina* | AM114726 | AM110253 |
| Arecales | Areaceae | *Phoenix dactylifera* | GU811709 | GU811709 |
| Commelinales | Commelinaceae | *Tradescantia ohiensis* | HQ180889 | HQ182454 |
| Asparagales | Alliaceae | *Allium cepa* | JQ276391 | JQ273896 |
|  | Amaryllidaceae | *Amaryllis belladonna* | JQ276394 | JQ273899 |
|  | Asparagaceae | *Asparagus officinalis* | HQ180855 | HQ182417 |
|  | Asteliaceae | *Astelia alpina* | HM640648 | HM640530 |
|  | Themidaceae | *Bessera elegans* | HM640637 | HM640519 |
|  | Blandfordiaceae | *Blandfordia punicea* | HM640650 | HM640532 |
|  | Boryaceae | *Borya septentrionalis* | HM640651 | HM640533 |
|  | Hyacinthaceae | *Bowiea volubilis* | JQ276408 | JQ273913 |
|  | Laxmanniaceae | *Cordyline australis* | JQ276414 | JQ273919 |
|  | Doryanthaceae | *Doryanthes palmeri* | JQ276406 | JQ273911 |
|  | Hemerocallidaceae | *Hemerocallis minor* | HM640655 | HM640537 |
|  | Iridaceae | *Iris virginica* | HQ180869 | HQ182432 |
|  | Ixioliriaceae | *Ixiolirion tataricum* | HM640660 | HM640543 |
|  | Lanariaceae | *Lanaria lanata* | AY368376 | Z77313 |
|  | Tecophilaceae | *Tecophilaea cyanocrocus* | HM640661 | HM640544 |
|  | Xanthorrhoeaceae | *Xanthorrhoea preissii* | JQ276430 | JQ273935 |
|  | Agavaceae | *Yucca filamentosa* | HM640596 | HM640482 |
|  | Ruscaceae | *Polygonatum inflatum* | HM640570 | HM640456 |
|  |  | *Ruscus aculeatus* | JQ276421 | JQ273926 |
|  | Xeronemataceae | *Xeronema callistemon* | JQ276431 | JQ273936 |
|  | Hypoxidaceae | *Empodium veratrifolium* | AY368374 | Y14987 |
|  |  | *Hypoxis leptocarpa* | AY368375 | Z73702 |
|  |  | *Molineria capitulata* | HQ180860 | HQ182423 |
|  |  | *Rhodohypoxis milloides* | AY368377 | Z77280 |
|  | Apostasioideae | *Apostasia wallichii* | AY557212 | HQ182416 |
|  |  | *Neuwiedia veratrifolia* | AY557211 | AF074200 |
|  | Vanilloideae | *Cleistes rosea* | AJ310006 | AF074128 |
|  |  | *Pogonia ophioglossoides* | AJ310055 | AF074221 |
|  |  | *Vanilla planifolia* | AF263687 | AF074242 |
|  | Cypripedioideae | *Cypripedium passerinum* | AF263649 | AF074142 |
|  |  | *Paphiopedilum delenatii* | AY368379 | AF074208 |
|  |  | *Phragmipedium schlimii* | AY368380 | AF074213 |
|  |  | *Selenipedium chica* | AY368381 | AF074227 |
|  | Orchidoideae | *Altensteinia fimbriata* | AM900801 | FJ571315 |
|  |  | *Altensteinia paleacea* | AJ309989 | AF074105 |
|  |  | *Aulosepalum tenuiflorum* | AM884249 | AJ542433 |
|  |  | *Beloglottis costaricensis* | AJ543920 | AJ542432 |
|  |  | *Bipinnula fimbriata* | FR832064 | FR831958 |
|  |  | *Chloraea sp.* | AJ310005 | AF074125 |
|  |  | *Codonorchis sp.* | AJ310007 | AY368338 |
|  |  | *Corybas diemenicus* | AJ310010 | AF074135 |
|  |  | *Cranichis fertilis* | AJ310013 | AF074137 |
|  |  | *Cryptostylis subulata* | AJ310015 | AF074140 |
|  |  | *Cyanicula gemmata* | AJ310017 | AY381116 |
|  |  | *Cyclopogon epiphyticus* | AJ543927 | AJ542425 |
|  |  | *Dichromanthus aurantiacus* | AJ543913 | AJ542439 |
|  |  | *Disa glandulosa* | AF263654 | AF274006 |
|  |  | *Disperis lindleyana* | AY370652 | AY370651 |
|  |  | *Diuris sulphurea* | AF263655 | AF074152 |
|  |  | *Dossinia marmorata* | AJ543947 | AJ542405 |
|  |  | *Eltroplectris calcarata* | AJ519450 | AJ519446 |
|  |  | *Eriochilus cucullatus* | AJ310028 | AF074166 |
|  |  | *Eurystyles borealis* | AJ543925 | AJ542427 |
|  |  | *Funkiella hyemalis* | AJ543923 | AJ542429 |
|  |  | *Gavilea venosa* | FR832096 | FR831991 |
|  |  | *Gennaria diphylla* | AY368383 | AY368341 |
|  |  | *Geoblasta penicillata* | FR832098 | FR831993 |
|  |  | *Gonatostylis vieillardii* | AJ310034 | AY381122 |
|  |  | *Goodyera pubescens* | AF263663 | AF074174 |
|  |  | *Habenaria repens* | AJ310036 | AF074177 |
|  |  | *Herschelianthe spathulata* | AY368384 | AY368342 |
|  |  | *Ludisia discolor* | AJ543911 | AJ542395 |
|  |  | *Mesadenus lucayanus* | AJ543916 | AJ542436 |
|  |  | *Megastylis rarus* | AJ310044 | AY381126 |
|  |  | *Microthelys aff. Minutiflora* | AJ543922 | AJ542430 |
|  |  | *Microtis parviflora* | AJ310045 | AF074194 |
|  |  | *Monadenia* | AJ310047 | AY368344 |
|  |  | *Odontorrhynchus variabilis* | AJ543926 | AJ542426 |
|  |  | *Pachyplectron arifolium* | AJ310051 | AF074205 |
|  |  | *Platanthera ciliaris* | AF263678 | AF074215 |
|  |  | *Platythelys querceticola* | AY368386 | AF074216 |
|  |  | *Ponthieva racemosa* | AJ310056 | AF074223 |
|  |  | *Pterostylis nutans* | GQ866247 | AF074224 |
|  |  | *Pterygodium catholicum* | HQ438200 | AY368346 |
|  |  | *Sarcoglottis acaulis* | AJ310068 | FJ571350 |
|  |  | *Spiranthes cernua* | AF263682 | AF074229 |
|  |  | *Stenoglottis longifolia* | AY368387 | AY368349 |
|  | Epidendroideae | *Acanthephippium mantinianum* | AF263618 | AF074100 |
|  |  | *Acineta chrysantha* | AF263619 | AF074102 |
|  |  | *Aeranthes ramosa* | AY368390 | AF074104 |
|  |  | *Agrostophyllum majus* | AY368391 | AF518054 |
|  |  | *Ancistrochilus rothschildianus* | EF079334 | AF264152 |
|  |  | *Angraecum sesquipedale* | AF263621 | AF074106 |
|  |  | *Aplectrum hyemale* | EU266416 | AF074108 |
|  |  | *Appendicula cornuta* | AY368393 | AY368352 |
|  |  | *Arpophyllum giganteum* | AF265485 | AF074110 |
|  |  | *Arundina graminifolia* | AF302692 | AF074111 |
|  |  | *Aspasia lunata* | EF079200 | FJ534160 |
|  |  | *Basiphyllaea corallicola* | AY121722 | AF264155 |
|  |  | *Bletia rosea* | AF263629 | AF264157 |
|  |  | *Bletilla striata* | EF079331 | AF074114 |
|  |  | *Bulbophyllum lobbii* | AY368395 | AF074115 |
|  |  | *Calypso bulbosa* | EF525689 | AF074120 |
|  |  | *Caularthron bilamellatum* | AF263780 | AF518059 |
|  |  | *Cephalanthera longibracteata* | HM640666 | HM640549 |
|  |  | *Chysis bractescens* | EF079351 | AF074126 |
|  |  | *Coelia triptera* | EF079353 | AF074132 |
|  |  | *Coelogyne cristata* | AF302707 | AF074133 |
|  |  | *Collabium sp.* | AF263645 | AF264163 |
|  |  | *Coryanthes verrucolineata* | AY368398 | AF074134 |
|  |  | *Cryptocentrum peruvianum* | DQ210786 | AF074139 |
|  |  | *Diaphananthe rutila* | AY368403 | AF074147 |
|  |  | *Dipteranthus grandiflorus* | AY368405 | AY368357 |
|  |  | *Dracula chimaera* | AF265444 | AF518039 |
|  |  | *Epipactis helleborine* | AF263659 | Z73707 |
|  |  | *Eriopsis biloba* | DQ210866 | AF074167 |
|  |  | *Eulophia streptopetala* | EF079258 | AM235039 |
|  |  | *Listera smallii* | AF263668 | AF074184 |
|  |  | *Nervilia* | AY368420 | AF074199 |
|  |  | *Palmorchis trilobulata* | AJ310052 | AF074206 |
|  |  | *Sobralia macrantha* | AF263681 | AF074228 |
|  |  | *Calanthe calanthoides* | AF263632 | AF264159 |
|  |  | *Cattleya dowiana* | AF263638 | AF074122 |
|  |  | *Cymbidium ensifolium* | AF263648 | AF074141 |
|  |  | *Dendrobium kingianum* | AF263651 | AF074146 |
|  |  | *Dendrobium moniliforme* | AB519774 | AB519788 |
|  |  | *Dendrobium nobile* | AB519772 | AB519785 |
|  |  | *Dendrobium pulchellum* | AB519778 | AB519789 |
|  |  | *Dendrobium tosaense* | AB519770 | AB519782 |
|  |  | *Dichaea riopalenquensis* | EU123657 | AF074149 |
|  |  | *Earina autumnalis* | EF079336 | AF074155 |
|  |  | *Encyclia oncidioides* | AF263788 | AF518062 |
|  |  | *Eria ferruginea* | AF263660 | AF074164 |
|  |  | *Galeandra devoniana* | AY368408 | AF074171 |
|  |  | *Gomesa flexuosa* | FJ565141 | FJ534252 |
|  |  | *Gongora amparoana* | AY368409 | AY368358 |
|  |  | *Grandiphyllum auriculum* | FJ565155 | FJ534200 |
|  |  | *Helcia brevis* | EF079229 | FJ534148 |
|  |  | *Helleriella guerrerensis* | AF263761 | AF518029 |
|  |  | *Hintonella mexicana* | FJ564940 | FJ534195 |
|  |  | *Holcoglossum flavescens* | EU558965 | HQ404489 |
|  |  | *Ionopsis minutiflora* | FJ565047 | FJ534204 |
|  |  | *Isochilus amparoanus* | AY368412 | AY368361 |
|  |  | *Leochilus leiboldi* | FJ564765 | FJ534194 |
|  |  | *Liparis lilifolia* | AF263667 | AF074183 |
|  |  | *Lycaste* | AF263669 | AF074185 |
|  |  | *Lycomormium squalidum* | AY368414 | AF074186 |
|  |  | *Macradenia rubescens* | FJ564839 | FJ534181 |
|  |  | *Masdevallia uniflora* | AF265446 | AF518040 |
|  |  | *Maxillaria cucullata* | DQ210753 | AF074190 |
|  |  | *Meiracyllium trinasutum* | EF079317 | AF074192 |
|  |  | *Neofinetia falcata* | EF655782 | AF074197 |
|  |  | *Octomeria gracilis* | AF265484 | AY368365 |
|  |  | *Odontoglossum sanguineum* | FJ564985 | FJ534145 |
|  |  | *Oeceoclades saundersiana* | AY368422 | AY368366 |
|  |  | *Oncidium Gower Ramsey* | GQ324949 | GQ324949 |
|  |  | *Phalaenopsis* | AF263677 | AY916449 |
|  |  | *Pleione formosana* | AF263679 | AF264173 |
|  |  | *Psychopsis limminghei* | FJ565152 | FJ534254 |
|  |  | *Restrepia xanthophthalma* | AY370654 | AY370653 |
|  |  | *Rossioglossum ampliatum* | FJ563834 | FJ534135 |
|  |  | *Saundersia paniculata* | FJ564734 | FJ534155 |
|  |  | *Stanhopea* | AY368430 | AF074230 |
|  |  | *Tolumnia gundlachii* | FJ565132 | FJ534250 |
|  |  | *Trichocentrum stipitatum* | FJ565138 | FJ534251 |
|  |  | *Trichopilia subulata* | FJ565033 | FJ534153 |
|  |  | *Zygopetalum intermedium* | AF263689 | AF074246 |
