# Supplementary material for: Evolution and Biogeography of the Slipper Orchids: Eocene Vicariance of the Conduplicate Genera in the Old and New World Tropics
Source: PLoS One. 2012 Jun 7;7(6):e38788. doi: 10.1371/journal.pone.0038788 (PMC3369861; doi:10.1371/journal.pone.0038788)
Supplement: Table S4 — Amplification results of the ndh F gene with different primer pairs in the present study. (DOC) [file pone.0038788.s008.doc]

**Table S4. Amplification results of the *ndh*F gene with different primer pairs in the present study.**

| **Genera** | ***trn*N+*trn*L** | ***ndh*FcF+*ndh*FaR** |
| --- | --- | --- |
| ***Cypripedium*** | ~ 6000 bp or multiple weak bands | ~ 1700-2200 bp or failed |
| ***Selenipedium*** | ~ 6000 bp | ~ 1700 bp |
| ***Mexipedium*** | ~ 1400 bp | failed |
| ***Phragmipedium*** | ~ 1400-1600 bp | failed |
| ***Paphiopedilum*** | multiple weak bands or failed, not further analyzed | multiple weak bands or failed, not further analyzed |
